# Rapid Column-Free Enrichment of Mononuclear Cells from Solid Tissues

**DOI:** 10.1038/srep12490

**Published:** 2015-07-30

**Authors:** Steven D. Scoville, Karen A. Keller, Stephanie Cheng, Michael Zhang, Xiaoli Zhang, Michael A. Caligiuri, Aharon G. Freud

**Affiliations:** 1 Medical Scientist Training Program, College of Medicine, The Ohio State University, Columbus, Ohio 43210, USA; 2 Division of Hematology, Department of Internal Medicine, The Ohio State University, Columbus, Ohio 43210, USA; 3 Division of Hematopathology, Department of Pathology, The Ohio State University, Columbus, Ohio 43210, USA; 4 Center for Biostatistics, The Ohio State University, Columbus, Ohio 43210; 5 The Ohio State University Comprehensive Cancer Center, The James Cancer Hospital & Solove Research Center, Columbus, Ohio 43210, USA

## Abstract

We have developed a rapid negative selection method to enrich rare mononuclear cells from human tissues. Unwanted and antibody-tethered cells are selectively depleted during a Ficoll separation step, and there is no need for magnetic-based reagents and equipment. The new method is fast, customizable, inexpensive, remarkably efficient, and easy to perform, and per sample the overall cost is less than one-tenth the cost associated with a magnetic column-based method.

Investigation of the human immune system typically involves the isolation of viable leukocyte populations from various tissues. Even without complete purification, an enrichment step via positive or negative selection is usually necessary in order to optimize assay signal-to-noise ratios and to reliably perform immunophenotyping by flow cytometry. This is particularly true when studying rare populations such as CD34(+) hematopoietic progenitor cells, minor T cell subsets, and the recently described heterogeneous population of non-T, non-B innate lymphoid cells (ILC), which are scarce in peripheral blood (PB) yet relatively enriched in secondary lymphoid tissues (SLT)^1^,^2^,^3^,^4^,^5^,^6^.

Commonly used methods of cell separation include fluorescence activated cell sorting (FACS) and magnetic-based selection techniques. For these approaches, tissue-derived single-cell suspensions are stained with either fluorescent molecule- or magnetic particle-conjugated antibodies that enable high specificity for positive and/or negative selection. Although cell sorting is essentially the gold standard for cell purification, its utility as a sole means of purifying or even enriching rare populations can be impractical due to the large lengths of time, equipment and sorting expenses, and required technical expertise associated with this approach. Therefore, FACS-based purification is typically preceded by other enrichment methods. Magnetic-based methods in particular are very effective for pre-FACS cell enrichment, and we previously developed and utilized a magnetic column-based method (MCM) to enrich rare natural killer (NK) cells and other ILC from SLT^4^,^7^. However, the magnetic based system is limited by both financial and time related constraints. Magnetic, antibody-conjugated beads, columns, and magnets represent considerable recurring expenses associated with this approach. Column purification often represents the rate limiting enrichment step, most noticeable when columns clog and/or the number of samples supersedes the available number of magnets, necessitating multiple rounds of magnetic selection. Finally, recent reports have indicated that magnetic selection through columns can even interfere with downstream functional assays^8^. Thus, we proceeded to pursue an alternative method for enrichment.

A widely used alternative to FACS and magnetic enrichment strategies utilizes a bivalent antibody reagent, such as RosetteSep (StemCell Technologies), for negatively enriching mononuclear cell populations from tissues such as PB, umbilical cord blood, and bone marrow^9^,^10^. One end of the bivalent antibody is specific for glycophorin A, expressed on human red blood cells (RBC), and the other side is variable and may be directed against a number of available lineage (Lin)-specifying antigens (e.g. CD3 or CD19 on T or B lymphocytes, respectively). Depending upon the target cell to be enriched by negative selection, a cocktail of bivalent non-target cell directed antibodies is added to the liquid tissue specimen and results in tethering RBC to the non-target populations. During subsequent Ficoll-based density centrifugation separation, the non-target, RBC-coated populations are then pulled into the RBC pellet at the bottom of the tube, whereas the unlabeled, uncoated target cells of interest are enriched and remain at the mononuclear interface layer above the Ficoll.

Given the necessity for RBC to be present for the bivalent antibody reagent to work, solid tissues such as SLT are not inherently amenable to this separation approach. Nonetheless, we reasoned that if we could provide an exogenous source of RBC to SLT-derived single-cell suspensions and thus make a transiently RBC-rich, liquid sample, we could theoretically utilize this reagent to rapidly enrich rare populations. To test this hypothesis, we directly compared our previously published MCM to a new bivalent antibody-based method (BAM) for the enrichment of rare Group 3 ILC (ILC3) and NK cells from pediatric tonsils. Figure 1 shows a schematic representation of the two enrichment methods (see also **online Methods**). As depicted, the MCM involves a Ficoll separation step followed by T and B cell depletion using magnetically-labeled antibodies against CD3 and CD19, respectively. For the BAM, single-cell suspensions are first mixed with a combination of allogeneic, leukocyte-depleted RBC and a bivalent antibody cocktail consisting of a mixture of bivalent antibodies against glycophorin A and CD3, CD4, CD19, CD36, CD66b, or CD123. This antibody cocktail effectively depletes B and T cells as well as granulocytes, monocytes, and dendritic cells (DC). However, ILC populations are not depleted, because none of these antigens are expressed on human ILC^11^ (S.D.S and A.G.F. unpublished observations).

Among unfractionated tonsil mononuclear cell preparations generated only by Ficoll density separation, most leukocytes are T or B lymphocytes, whereas ILC3 and NK cells each represent <1% of cells (Fig. 2a,b). However, following the MCM and BAM, T and B cells are significantly depleted, and ILC3 and NK cells are significantly enriched (Fig. 2a,b) (n = 5). Of note, whereas other CD3(-)CD19(-) cell types, such as monocytes and DC, are also enriched with the MCM, these cells are effectively depleted using the BAM as expected (data not shown). We did not observe statistically significant differences between the enrichment methods in terms of absolute numbers of recovered ILC3 and NK cells (Fig. 2b). However, we noticed a trend that cells isolated with the MCM, compared to the BAM, were more activated. This difference was especially noticeable when comparing the percentage of unstimulated ILC3 cells that produced IL-22 and NK cells that produced IFN-γ between the two methods (Fig. 2c).

In order to test if the BAM could be used to enrich other populations from SLT, we used a similar bivalent antibody cocktail specifically for the enrichment of human monocytes and CD8+ T cells on a fresh tonsil specimen. Similar to the enrichment of ILC3 and NK cells described above, we observed approximately 10-fold enrichment of monocytes and CD8+ T cells with the use of their respective enrichment cocktails (Fig. 2d). Collectively, these data suggest that potentially any mononuclear cell population could be enriched using the BAM provided an appropriate cocktail of bivalent antibodies can be constructed.

Given the success of this novel approach in tonsils, we also investigated whether the BAM can be used to enrich rare leukocytes from other solid tissues. To date, we have successfully enriched NK cells from human thymus and lymph nodes using the BAM (data not shown). As this approach was successful in each of these cases, it is thus a valid alternative to previously used methods of leukocyte enrichment from secondary lymphoid tissues and other leukocyte predominant solid tissues. Although not tested here, this protocol may also have the potential to be applied in other non-lymphoid predominant tissues if appropriate depletion antibodies against the predominant mesenchymal and/or other unwanted components can be included. As such, this method may be especially beneficial to those comparing various immune subsets in the settings of diseased versus healthy solid tissues.

Lastly, we analyzed the core components of each method to compare the overall costs and time associated with enriching ILC3 and NK cells from 1E9 tonsil-derived cells, an approximate average tonsillar single cell suspension yield (Table 1 and data not shown). For the MCM, we included the costs of the CD3 and CD19 magnetically-labeled antibodies as well as the disposable magnetic depletion columns. For the BAM, we included the costs of the commercially available bivalent antibody cocktail together with the costs associated with obtaining leukocyte-depleted RBC. Remarkably, when just taking these costs into account we found that the BAM is over 10-fold less expensive compared to the MCM (Table 1). In addition, while not specifically quantified, we estimate that the time required to enrich ILC from an average-size tonsil specimen (3–5 g) using the BAM is 2–3 hr less than that associated with the MCM. In our laboratory, using the BAM, one trained individual can now easily process up to four tonsil specimens in 10 hours, whereas using the MCM limited us to processing two tonsils during the same period of time.

To date, our laboratory has used the MCM and BAM approaches to process >700 tonsils and >300 tonsils, respectively. The BAM protocol is simple to use and has greatly benefited our laboratory in reducing overall costs in terms of both personnel time and consumables, at an estimated savings of greater than $250,000 U.S. dollars in just over 1 year. Therefore, given the significant reductions in supply and labor costs associated with the BAM together with the evidence for reduced baseline leukocyte activation, we feel that this new method provides significant advantages over the MCM and can be useful to a very broad group of investigators interested in enriching different target cell populations.

## Methods

### Tissue procurement

All protocols were approved by the Institutional Review Board (IRB) of The Ohio State University (OSU) and performed in accordance with approved guidelines. Fresh human pediatric tonsils were obtained through the National Cancer Institute approved Cooperative Human Tissue Network (CHTN) from Nationwide Children’s Hospital, Columbus, Ohio as performed previously^12^. Donor consent was obtained where applicable and under accordance with our approved OSU IRB protocol. Single cell suspensions were generated with the gentleMACS tissue dissociator (Miltenyi Biotec) using C-tubes (Miltenyi Biotec) and the pre-programmed “spleen 1” setting. Tonsil single cell suspensions were split in half and processed using either the MCM or the BAM (see below). Allogeneic leukocyte-reduced (<5E6 leukocytes per unit, ~300 mL each) human AB Rh+ RBC units were purchased from the American Red Cross, Columbus, Ohio. Residual leukocytes were depleted by diluting the RBC 1:2 in fetal bovine serum (FBS, Life Technologies), centrifuging the diluted RBC at 754 × *g* for 30 min at room temperature (RT) with the brake off, and then aspirating off the supernatants to remove any residual leukocytes. Resultant leukocyte-depleted RBC preparations were tested by flow cytometry to confirm the absence of residual leukocytes, and no CD45+ leukocytes were detected.

### Cell Enrichment Techniques

#### Magnetic column-based method

**Figure 1:**
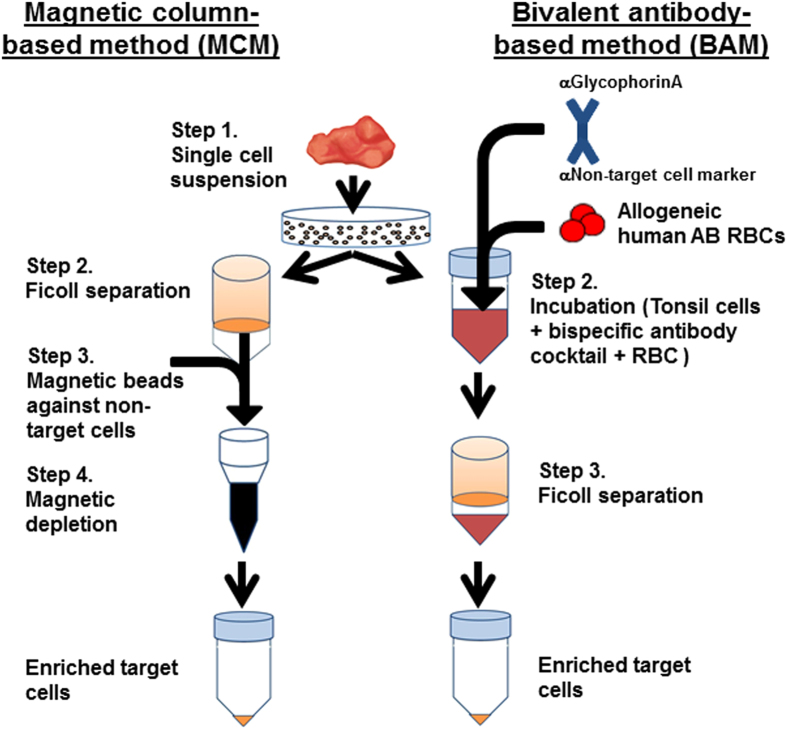
Schematic representations of the MCM and BAM. As depicted on the left with the MCM, single cell suspensions are first layered over Ficoll to obtain mononuclear cells, and then the latter are depleted of T- and B-cells with CD3 and CD19 microbeads, respectively, and magnetic depletion columns. As depicted on the right with the BAM, single cell suspensions are first incubated with allogeneic leukocyte-depleted RBC and the bivalent antibody cocktail followed by Ficoll density centrifugation separation. Enriched target mononuclear cells are then obtained from the Ficoll/supernatant interface. We thank Ashley Smith for her illustrative assistance with this figure.

The MCM is based on our previously published protocol^7^ (Fig. 1). For every 0.5 g of tonsil tissue, single cell preparations were diluted in 25 mL room temperature phosphate-buffered saline (PBS, Gibco) and layered over 15 mL of Ficoll-Paque^TM^ PLUS (GE Healthcare) in a 50 cc polypropylene centrifuge tube (Corning). Tubes were then spun at 754 × *g*, RT, for 30 min with the break off. The mononuclear layers at the Ficoll-supernatant interface were obtained by manual pipetting, washed in PBS, combined into a single tube, and then enumerated by trypan blue dye exclusion using a hemacytometer. Mononuclear cell preparations were depleted of CD3+ and CD19+ cells using MACS reagents (Miltenyi Biotec) following the manufacturer’s suggested protocol. Briefly, the cells were incubated for 30 min at 4 °C at a concentration of 1E7 cells per 100 μL consisting of 20 μL each of CD3 and CD19 microbeads (Miltenyi Biotec) plus 60 μL “PBS+” [PBS with the addition of 0.8% EDTA (4 mM pH 8.0) and 1% FBS (Sigma Aldrich)]. Post incubation, cells were washed with PBS+, resuspended at a concentration of 3.3E7 cells/mL PBS+, filtered over a 70 μm nylon membrane (Corning), and passed over LD depletion columns (Miltenyi Biotec) at 3 mL per column. Columns were each washed twice with 3 mL of PBS+. The eluted, CD3/CD19-depleted, ILC/NK-enriched cell fractions were enumerated and used for flow cytometry immunophenotyping and/or *in vitro* stimulation for cytokine production (see below).

#### Bivalent antibody-based method

Tonsil-derived single cell suspensions were pelleted by centrifugation at 524 × *g* for 10 min at RT. The cell pellets were then resuspended in 2 mL RT FBS, 1 mL leukocyte-depleted RBC, and 100 μL RosetteSep “Human NK cell Enrichment Cocktail” (StemCell Technologies) per 1E8 cells. The RosetteSep “Human NK cell Enrichment Cocktail” contains bivalent antibodies to tether cells expressing CD3, CD4, CD14, CD19, CD36, CD66b, and CD123 to human RBC. Where indicated, we also used the human CD8+ T cell and monocyte enrichment cocktails (StemCell Technologies). The tonsil cell/FBS/RBC/RosetteSep mixtures were incubated at RT for 30 min on a nutator. After incubation, the mixtures were diluted 2-fold in RT PBS and then layered over Ficoll (25 mL of cell mixture over 15 mL Ficoll in each 50 cc tube) and centrifuged at 754 × *g* for 30 min at RT with the brake off. Mononuclear cell layers at the Ficoll/supernatant interface were collected, and then residual RBC were lysed by resuspending the mononuclear cell preparations in 10 mL 0.8% NH_4_Cl buffer (StemCell Technologies) per donor for 5–10 min at RT. Enriched cells were then washed with PBS, enumerated as above, and used for flow cytometry immunophenotyping and/or *in vitro* stimulation for cytokine production (see below).

### Flow Cytometry

Flow cytometry was performed on 1E6 stained cells from each enriched fraction, including a non-enriched sample from each tonsil for comparison. Surface and intracellular flow cytometry was performed as previously described^12^ on a Becton Dickenson LSR II flow cytometer using FACS Diva analysis software (BD Biosciences). The following commercially available antibodies were used: CD8-PE, CD19-PE, CD19-FITC, CD94-APC, CD3-APC-H7, and IFN-γ-BV421 (BD Biosciences); CD3-PerCP-Vio700, CD14-PerCP-Vio700, CD117-PE-Vio770, CD3-VioGreen, and CD14-VioGreen (Miltenyi Biotec); and IL-22-PE (R&D Systems). Cytofix/Cytoperm and PermWash reagents (BD Biosciences) were used to perform intracellular flow cytometry as previously described^12^.

### *In vitro* stimulation of enriched ILC for cytokine production

Enriched ILC were stimulated *in vitro* for 6 hr with a combination of 81 nM phorbol 12-myristate 13-acetate and 1.34 μM ionomycin (eBioscience) plus 1 nM recombinant human IL-2 (Peprotech). Brefeldin A (BD Biosciences) was added to the cultured cells during the final 4 hr of stimulation, and then the cells were harvested and analyzed by intracellular flow cytometry for the production of IFN-γ and IL-22.

### Statistical analyses

Linear mixed effects models were used for analysis of correlation among observations from the same donor. The differences between different conditions were estimated from those models. Holm’s procedure was used to adjust for multiple comparisons. After adjustment for multiple comparisons, p-values < 0.05 were considered significant. Summary statistics, such as mean and standard deviation, were provided for all variables. SAS 9.3 was used for analysis (SAS Institute Inc., NC).

## Additional Information

**How to cite this article**: Scoville, S. D. *et al*. Rapid Column-Free Enrichment of Mononuclear Cells from Solid Tissues. *Sci. Rep*. **5**, 12490; doi: 10.1038/srep12490 (2015).

## Figures and Tables

**Figure 2:**
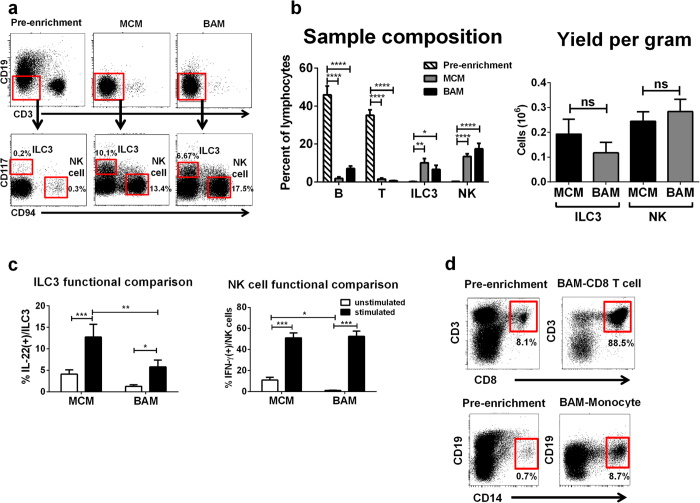
Comparison of the MCM and BAM. (**a**) Flow cytometry of pre-enriched (left column) versus MCM-enriched (middle column) versus BAM-enriched (right column) ILC populations from a representative donor. (**b**) Pre and post enrichment composition comparison (left) and assessment of total yields of ILC3 and NK cells per gram of tonsil (right). non- significant (ns) (n = 5). (**c**) Cytokine production by enriched ILC3 and NK cells following MCM and BAM enrichment. White boxes indicate unstimulated, black boxes indicate stimulated samples (n = 6). (**d**) CD8+ T cell and CD14+ monocyte enrichments from a pediatric tonsil using the corresponding bivalent antibody cocktails. *p < 0.05; **p < 0.01; ***p < 0.001; ****p < 0.0001. (**b**,**c**) indicates data from 2 independent experiments with at least two donors. Linear mixed effects models were used for statistical comparison and adjusted for multiple comparisons using Holm’s procedure. Error bars indicate s.e.m.

**Table 1:** Cost and time comparison between the MCM and BAM.

Method	Reagent	Unit Price	Amount Required	Price per 1E9 cells
BAM	RBC unit	$1.68/mL	10 mL	$16.77
	FBS	$0.77/mL	20 mL	$15.44
	RosetteSep	$59.30/mL	1 mL	$59.30
	Total			$91.51
	Estimated processing time*			2.5 hr
MCM	CD3 microbeads	$615.00/vial	1 vial	$615.00
	CD19 microbeads	$620.00/vial	1 vial	$620.00
	LD columns	$14.40/column	10 columns	$144.00
	Total			$1379.00
	Estimated processing time*			5 hr

^*^The depicted time is estimated based on our experience processing one average size pediatric tonsil (3–5 g), which yields approximately 1E9 total single cells prior to enrichment. Note that with more tonsils, the processing time per tonsil with the MCM non-linearly increases if the number of available magnets is limiting.
